# Characteristics and outcomes of therapy-related acute leukemia following autologous transplant (Auto-HCT) for multiple myeloma

**DOI:** 10.1038/s41409-024-02455-4

**Published:** 2024-10-30

**Authors:** Omar Elghawy, Saarang Deshpande, Jonathan Sussman, Alfred Garfall, Adam Cohen, Shivani Kapur, Sandra Susanibar-Adaniya, Dan Vogl, Adam Waxman, Edward Stadtmauer

**Affiliations:** 1 Division of Hematology Oncology, Department of Medicine, University of Pennsylvania, Philadelphia, PA USA; 2 Abramson Cancer Center, University of Pennsylvania, Philadelphia, PA USA

**Keywords:** Stem-cell therapies, Disease-free survival

## Abstract

With a prolonging duration of survivorship, patients with multiple myeloma (MM) who receive high-dose chemotherapy and autologous hematopoietic stem cell transplantation (auto-HCT) have an increased risk of secondary malignancy, most concerning acute leukemia. We retrospectively reviewed the records of all patients with MM who underwent auto-HCT between January 1, 2010, and January 1, 2023, who later developed therapy-related acute leukemia (t-AL). Of 1770 patients with MM who underwent auto-HCT, 18 (1.01%) developed t-AL at a mean interval of 60.0 ± 41.3 months after auto-HCT. The patients with t-AL consisted of 9 (50%) with B-cell acute lymphoblastic leukemia (B-ALL), 8 (44.4%) with acute myeloid leukemia (AML), and 1 (5.6%) with acute promyelocytic leukemia (APML). All patients had received an alkylating agent as part of induction, and the majority received lenalidomide as maintenance therapy. Genetic abnormalities of t-AL were consistent with prior reports. Median overall survival from diagnosis of t-AL was 19.5 months. In patients with t-AL who entered CR, long term survival was common. Further research on predisposing conditions to developing t-AL in patients with MM undergoing auto-HCT is warranted.

## Introduction

Multiple myeloma (MM) is a clonal plasma cell proliferative disorder that represents 10% of all hematological malignancies and is responsible for 20% of deaths from hematological malignancies [1, 2]. Multiple novel therapies including immunomodulators, proteasome inhibitors, and CAR-T therapy have resulted in a significant improvement in the survival of MM patients [3–5]. Despite this, myeloma remains the leading indication for autologous stem-cell transplantation (ASCT) which is considered to be the standard of care for these patients [6, 7]. While PFS and OS for MM have drastically improved with utilization of ASCT, treatment-related complications such as infections and organ system failure remain a persistent challenge [7–9].

Treatment-related acute leukemia (t-AL) is a well-recognized complication occurring following exposure to cytotoxic chemotherapy or radiotherapy including in those who receive ASCT [10]. The cumulative risk of t-ALs for MM patients receiving ASCT has varied widely from 1.1% to up to 24.3% after ASCT [11, 12]. Several chemotherapeutic agents have been associated with the development of t-ALs in MM patients including lenalidomide and etoposide [13]. The development of t-AL has been associated with considerable morbidity and poor survival due to high-risk cytogenetics and frequent TP53 mutations [14–16]. Many of these studies however have been conducted prior to the advent of several new chemotherapeutics for the treatment of acute leukemias which have revolutionized survival in these patients. This study aims to investigate the clinical characteristics and treatment outcomes of patients who developed t-AL after ASCT for MM

## Methods

A single-institution study of patients with a diagnosis of multiple myeloma who subsequently received an autologous transplant from January 1, 2010, to January 1, 2023, was performed via query of the University of Pennsylvania Cancer Registry. All patient charts were manually screened and patients who subsequently developed acute lymphoid or myeloid leukemia as defined by the 2016 World Health Organization classification were included within this study [17]. Patients with a diagnosis of myelodysplastic syndrome who did not progress to acute leukemia were excluded from this study. Patient demographics, molecular diagnostics, treatments received, and clinical outcomes were obtained and reviewed from the electronic medical record in accordance with the University of Pennsylvania Institutional Review Board and the Declaration of Helsinki.

All statistical tests were conducted using R v4.3.2. Baseline characteristics were assessed via independent t-test, Fisher’s exact test, and 2-sided Pearson chi-square analysis as appropriate. Survival analysis was conducted using Kaplan Meier methodology. Overall survival (OS) and progression free survival (PFS) were censored at date of last follow-up. A p value of <0.05 was deemed significant.

## Results

At our institution, 1770 patients underwent their first autologous SCT for MM during the specified time period. Of this cohort, 18 (1.01%) developed acute leukemia at a mean interval of 60.0 ± 41.3 months after autologous SCT for MM. Only two patients received NGS testing prior to t-AL diagnosis with neither exhibiting clonal hematopoiesis. Of patients who developed acute leukemia, the median follow-up was 102 months (range 3.2-301.5 months). Baseline patient demographics are shown in Table 1. The majority were female (66.7%) and white (83%). All had an ECOG of 0 or 1. Most patients (83%) had IgG myeloma and 78% exhibited Kappa light chain predominance.

**Table 1:** Patient Demographics and disease characteristics within study population.

Characteristic		Value (%)
		*n* = 18
Sex		
Male		6 (33%)
Female		12 (67%)
Race		
White		15 (83%)
Black		3 (17%)
Age at First Diagnosis (mean ± SD)		62.7 ± 8.4 years
ECOG		
0		6 (33%)
1		12 (67%)
Myeloma Type		
IgG		15 (83%)
IgA		3 (17%)
Kappa/Lambda		
Kappa		14 (78%)
Lambda		4 (22%)
Stage (ISS)		
I	2 (11%)	
II	12 (67%)	
III	4 (22%)	
Pre-HCT Exposure		
Lenalidomide	18 (100%)	
Alkylating Agent	2 (11%)	
Topoisomerase	0 (0%)	
Leukemia Type		
AML	8 (44%)	
APML	1 (6%)	
B-ALL	9 (50%)	
Top Recurrent Mutations		
DNMT3A	6 (33%)	
TP53	5 (28%)	
ETV6	4 (22%)	
RUNX1	4 (22%)	
GATA2	3 (17%)	
NOTCH2	3 (17%)	

**Fig. 1:**
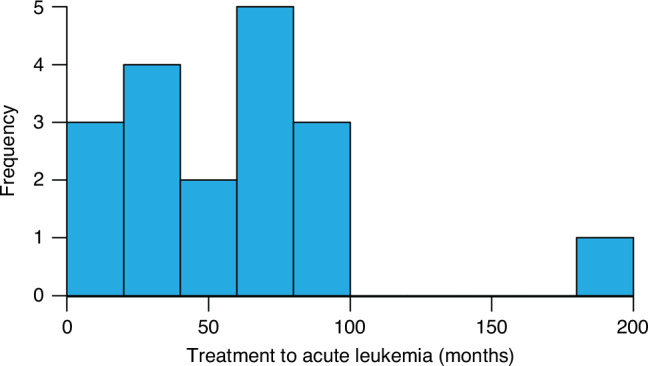
Histogram depicting time to diagnosis of post-transplant acute leukemia from time of ASCT.

Of patients who developed acute leukemia, 9 (50%) had B-cell acute lymphoblastic leukemia (B-ALL), 8 (44.4%) had acute myeloid leukemia (AML), and 1 (5.6%) developed acute promyelocytic leukemia (APML). The time from transplant to development of acute leukemia is depicted in Fig. 1. Median time to development of t-AL from transplant was 60.1 months (46.5 months for t-ALL and 74.7 months for t-AML). Treatment data prior to transplant is summarized in Table 2. Median lines of treatment prior to transplant was 1 (range 1-3). No patients received CAR T or bispecifics at any time point during their treatment course. All patients received cyclophosphamide for mobilization and melphalan conditioning. Twelve (67%) were exposed to maintenance lenalidomide with the remainder receiving bortezomib maintenance (6, 33%).

**Table 2:** Treatment overview of patients included within the study.

Characteristic	Value (%)
	*n* = 18
Treatment Lines prior to Transplant	
1	12 (67%)
2	4 (22%)
3	2 (11%)
First line treatment	
Lenalidomide, Bortezomib, and Dexamethasone	15 (83%)
Daratumumab, bortezomib, cyclophosphamide, and dexamethasone	3 (17%)
Second line treatment	
None	12 (67%)
Lenalidomide, Dexamethasone	3 (11%)
Carfilzomib, Lenalidomide and Dexamethasone	2 (11%)
Daratumumab, bortezomib, cyclophosphamide, and dexamethasone	1 (6%)
Third line Treatment	
None	16 (89%)
Pomalidomide, dexamethasone	2 (11%)
Stem Cell Mobilization	
Cytoxan	18 (100%)
Conditioning	
Melphalan	18 (100%)
GVHD	
No	17 (94%)
Yes	1 (6%)
Maintenance	
Lenalidomide	12 (67%)
Bortezomib	6 (33%)
Number of Treatments for Acute Leukemia	
1	9 (50%)
2	7 (39%)
3	2 (11%)
Acute Leukemia Remission	
No	8 (44%)
Yes	10 (56%)
Myeloma Remission	
No	3 (17%)
Yes	15 (83%)

The most frequently mutated genes via next-generation sequencing at the time of leukemia diagnosis were DNMT3A (6, 33%), TP53 (5, 28%), ETV6 (4, 22%), and RUNX1 (4, 22%). Two patients (11%) had next-generation sequencing prior to t-AL diagnosis without any alterations noted and the remainder (16, 89%) did not have next-generation sequencing performed prior to t-AL diagnosis. Three (37.5%) patients with AML had complex cytogenetics and two (22.2%) patients with B-ALL had BCR: ABL fusions. The one patient with APML had a positive 15:17 translocation.

Of the patients with AML, the majority (7/8, 87.5%) received azacitidine/venetoclax in the first line with the remaining patients receiving daunorubicin and cytarabine. Treatment for t-BALL was much more heterogeneous with 4 (44.4%) patients receiving mini-CVD with inotuzumab, 3(33.3%) patients receiving Hyper CVAD, 1 (11.1%) patient receiving VCR and 1 (11.1%) patient receiving Blinatumomab in the first line. The patient with APML was treated with arsenic and ATRA. No patients went on to receive allogeneic HCT as a treatment for their t-AL.

**Fig. 2:**
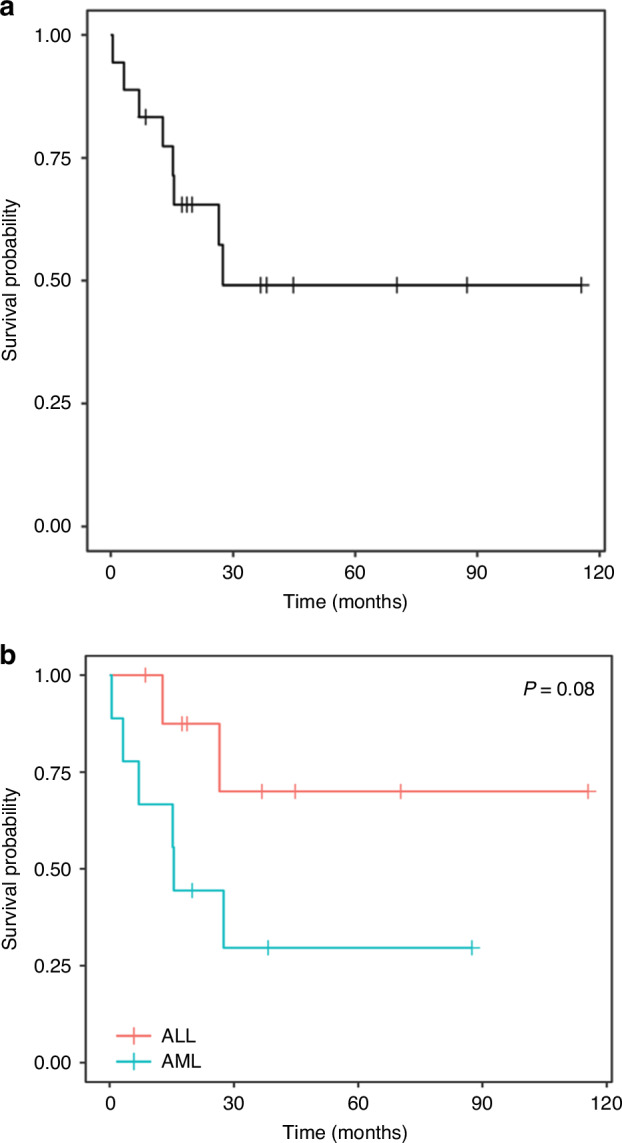
Overall survival from time of aute leukemia diagnosis. **a** Overall survival for the entire cohort calculated using Kaplan Meier methodology from time of acute leukemia diagnosis. **b** Overall survival for t-AML vs t-ALL calculated using Kaplan Meier methodology from time of acute leukemia diagnosis.

**Fig. 3:**
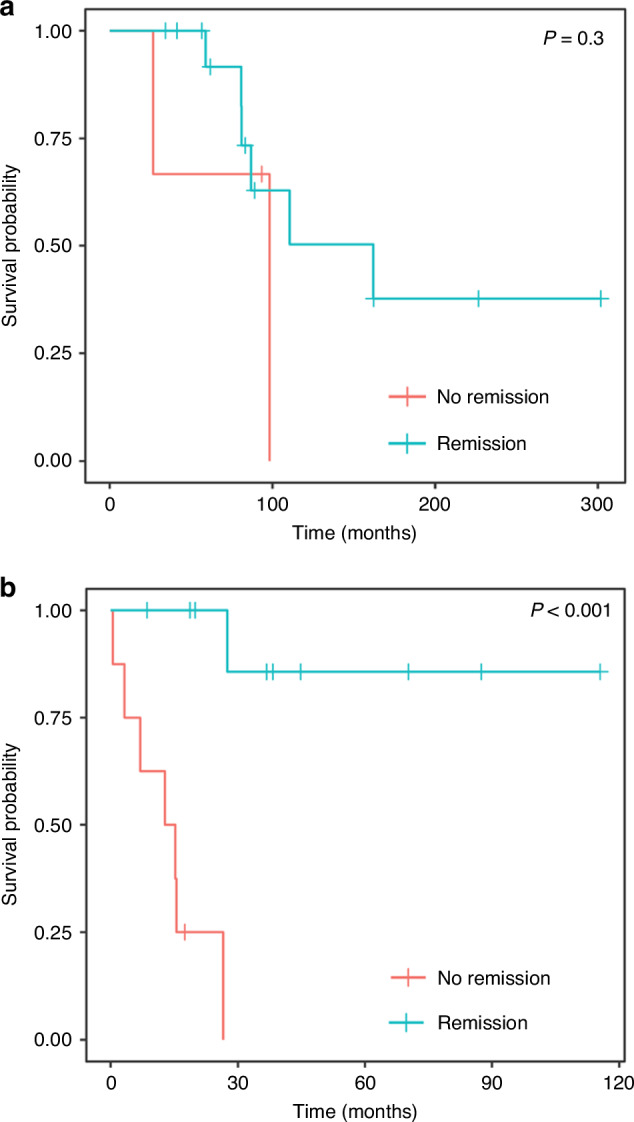
Kaplan Meier curves based on remission status at last follow-up. Redline represents patients with current disease at the time of last follow up while blue lines represent patients in remission at the time of last follow-up. **a** Kaplan Meier showing overall survival based on myeloma remission status. **b** Overall survival based on leukemia remission status.

Median overall survival for the entire cohort from time of tAL diagnosis was 19.5 months (Fig. 2a). Median OS for t-AML was shorter at 16.5 months compared to t-ALL (not reached) although this difference was not statistically significant (*p* = 0.08, Fig. 2b). At last follow up, 15 (83%) patients with tAL achieved MM remission, which was not associated with overall survival (8.19 years vs 10.50 years from time of transplant, *p* = 0.35; Fig. 3a) in this cohort. Two patients (11%) had myeloma relapse before tAL, and one patient (6%) had myeloma relapse 6 months after tAL. Ten patients were in acute leukemia remission at the time of the last follow-up. The presence of leukemia remission was significantly associated with overall survival (19.2 months vs mOS not reached *p* < 0.001, Fig. 3b).

## Discussion

In the current era of autologous SCT and many new anti-myeloma therapies, there is a longer duration of survivorship in MM and a higher likelihood of development of a second primary malignancy. Though the overall risk of development of a second primary malignancy is not higher than cohorts matched across demographic variables, there is an increased incidence of therapy-related myeloid malignancies after autologous SCT [18]. Our results further elaborate on the risk of t-AL development after autologous SCT for patients with MM.

In our cohort, we report a 1.01% risk of t-AL development after autologous SCT for MM which approximates prior reports. This is about half the reported incidence rate of 2% reported in a 2023 retrospective study at a median follow-up of four years [15]. A recent CIBMTR analysis demonstrated an observed-to-expected (O/E) ratio of 5.19 of AML development, significantly higher than the expected rate. This study only reported 8 AML or myelodysplastic syndrome (MDS) diagnoses in total for a cumulative incidence rate of 0.5% at 3 years and 1.51% at 7 years as well as only 3 incidences of ALL diagnoses post-transplant [18]. In the pivotal phase 3 DETERMINATION trial, second primary hematologic cancers occurred within 5-years in 13 (3.6%) patients in the transplantation 10 of which developed AML/MDS [19].

While our study did not specifically assess risk factors for the development of t-AL, prior studies have reported exposures that may increase risk. Older age at diagnosis appears to be a consistent risk factor across multiple studies for AML development [11, 15, 18]. Male sex and exposure to three or more prior chemotherapy regimens also appear to be risk factors for AML development [11, 18]. In this cohort, patients with t-ALL had worse overall survival than patients with t-AML, although not statistically significant. This is consistent with known outcomes measures for patients with primary ALL vs AML although a direct comparison in the post-transplant setting has not yet been established to the authors’ knowledge.

Multiple studies have also shown that exposure to alkylating agents is also associated with the subsequent development of acute leukemia across cancer types. Interestingly, all patients in our cohort had received cyclophosphamide as part of their mobilization regimen. Recent studies have shown that receipt of alkylating agents as induction for multiple myeloma is associated with increased incidence of tAL and inferior OS after t-AL diagnosis [15, 16]. Furthermore, in cohorts of patients with lymphoma who underwent autologous transplantation, those who received prolonged standard-dose alkylating agent therapy, rather than alkylating agents only during conditioning, demonstrated a significantly higher rate of t-MDS/AML development [20].

Receipt of lenalidomide also appears to be a significant risk factor for AML development. In our cohort, the majority of patients who developed t-AL have received lenalidomide maintenance therapy. Prior exposure to thalidomides is associated with *TP53* mutations in t-MDS/AML, and treatment with lenalidomide in vitro and in vivo provides a selective advantage to *Trp53*-mutant hematopoietic stem and progenitor cells providing a possible mechanistic reasoning for this increased incidence [21]. Lenalidomide has demonstrated PFS benefit in multiple landmark trials, namely CALGB100104 [22] and IFM2005-02 [23]; however, they both also demonstrated a numerically higher incidence of hematological malignancy (8 versus 1 and 13 versus 5, respectively) in the lenalidomide-treated arms versus control arms. In a patient-level meta-analysis including these trials, there was an increased incidence of hematologic second primary malignancy (5.3% for lenalidomide, 0.8% for control) with lenalidomide. Notably, the rate of progressive MM is far higher than the risk of second malignancy, and the time to progression of MM and OS was longer in lenalidomide arms. As well, time to death because of second primary malignancy or adverse event was no different between lenalidomide and control arms [24].

Notably, no patients within our cohort received certain immune therapeutics including bispecific antibodies and CAR-T cell therapy. Secondary malignancy is a well-recognized risk after commercial CAR T including CD19 and BCMA subtypes [25]. A recent large single-institution study showing that at a median follow-up of 10.3 months, 16/449 patients (3.6%) had a secondary primary malignancy [26]. These secondary malignancies range from secondary solid cancers, lymphomas, and acute leukemias and little is known regarding patients who may be at higher risk for the development of these cancers. As these therapies become more prevalent, the recognition and swift treatment of these cancers will become paramount.

Our analysis has multiple limitations. As a retrospective study, there is a risk of selection bias and potential incomplete data collection. The small number of patients who developed t-AL precluded analysis of outcomes outside of survival data. As our goal was to report the prevalence, molecular features, and treatment outcomes of patients developing t-AL, we did not perform any analysis to compare this group to those who did not develop t-AL as the small number of events would have underpowered such a design. Furthermore, it is difficult to directly compare our incidences and treatment outcomes for t-AL with prior studies given the constantly evolving treatment landscape for both multiple myeloma and acute leukemia. For instance, no patients within this cohort received bispecific antibodies or CAR-T therapy which are rapidly becoming integrated into the treatment paradigm for multiple myeloma. Conversely prior treatment lines associated with t-AL from much earlier studies of multiple myeloma were not represented within the patients within this study limiting our ability to directly compare treatment cohorts.

In summary, our results further elaborate on the risk of t-AL following autologous SCT for MM. We confirm a relatively rare incidence rate with poor outcomes in this population in those who did not reach CR. In patients with remission at last follow up however, long term survival is common. These findings will hopefully encourage further awareness of secondary malignancies in a population with prolonged survivorship durations.

## Data Availability

The datasets generated during and/or analyzed during the current study are available from the corresponding author on reasonable request.

## References

[1] Mikhael J, Bhutani M, Cole CE. Multiple myeloma for the primary care provider: a practical review to promote earlier diagnosis among diverse populations. Am J Med. 2023;136:33–41. 36150517 10.1016/j.amjmed.2022.08.030

[2] Cowan AJ, Green DJ, Kwok M, Lee S, Coffey DG, Holmberg LA, et al. Diagnosis and management of multiple myeloma: a review. JAMA. 2022;327:464–77. 35103762 10.1001/jama.2022.0003

[3] Ocio EM, Perrot A, Bories P, San-Miguel JF, Blau IW, Karlin L, et al. Efficacy and safety of isatuximab plus bortezomib, lenalidomide, and dexamethasone in patients with newly diagnosed multiple myeloma ineligible/with no immediate intent for autologous stem cell transplantation. Leukemia. 2023;37:1521–9. 37316728 10.1038/s41375-023-01936-7 PMC10264885

[4] Kaiser MF, Hall A, Walker K, Sherborne A, De Tute RM, Newnham N, et al. Daratumumab, Cyclophosphamide, Bortezomib, Lenalidomide, and Dexamethasone as induction and extended consolidation improves outcome in ultra-high-risk multiple myeloma. JCO. 2023;41:3945–55. 10.1200/JCO.22.02567 37315268

[5] Hansen DK, Sidana S, Peres LC, Colin Leitzinger C, Shune L, Shrewsbury A, et al. Idecabtagene Vicleucel for relapsed/refractory multiple myeloma: real-world experience from the myeloma CAR T Consortium. J Clin Oncol. 2023;41:2087–97. 36623248 10.1200/JCO.22.01365 PMC10082273

[6] Singh S, Sharma R, Singh J, Jain K, Paul D. Autologous stem cell transplantation for multiple myeloma in the novel agent era: Systematic review of Indian data and implications for resource constrained settings. J Cancer Res Ther. 2023;19:S12–19. 37147978 10.4103/jcrt.jcrt_503_22

[7] Jantunen E, Partanen A, Turunen A, Varmavuo V, Silvennoinen R. Mobilization strategies in myeloma patients intended for autologous hematopoietic cell transplantation. Transfus Med Hemother. 2023;50:438–47. 37899993 10.1159/000531940 PMC10603622

[8] Teh BW, Harrison SJ, Slavin MA, Worth LJ. Epidemiology of bloodstream infections in patients with myeloma receiving current era therapy. Eur J Haematol. 2017;98:149–53. 27717026 10.1111/ejh.12813

[9] Waszczuk-Gajda A, Penack O, Sbianchi G, Koster L, Blaise D, Reményi P, et al. Complications of autologous stem cell transplantation in multiple myeloma: results from the CALM study. J Clin Med. 2022;11:3541. 35743620 10.3390/jcm11123541 PMC9225651

[10] Patel SS, Rybicki LA, Corrigan D, Bolwell B, Dean R, Liu H, et al. Prognostic factors for mortality among day +100 survivors after allogeneic hematopoietic cell transplantation. Biol Blood Marrow Transpl. 2018;24:1029–34. 10.1016/j.bbmt.2018.01.016 PMC5953837 29369800

[11] Radivoyevitch T, Dean RM, Shaw BE, Brazauskas R, Tecca HR, Molenaar RJ, et al. Risk of acute myeloid leukemia and myelodysplastic syndrome after autotransplants for lymphomas and plasma cell myeloma. Leuk Res. 2018;74:130–6. 30055822 10.1016/j.leukres.2018.07.016 PMC6219911

[12] Taylor PR, Jackson GH, Lennard AL, Hamilton PJ, Proctor SJ. Low incidence of myelodysplastic syndrome following transplantation using autologous non-cryopreserved bone marrow. Leukemia. 1997;11:1650–3. 9324284 10.1038/sj.leu.2400795

[13] Pedersen-Bjergaard J, Andersen MK, Christiansen DH. Therapy-related acute myeloid leukemia and myelodysplasia after high-dose chemotherapy and autologous stem cell transplantation. Blood. 2000;95:3273–9. 10828005

[14] Kallen ME, Koka R, Singh ZN, Ning Y, Kocoglu MH, Badros AZ, et al. Therapy-related B-lymphoblastic leukemia after multiple myeloma. Leuk Res Rep. 2022;18:100358. 36353199 10.1016/j.lrr.2022.100358 PMC9637917

[15] Yalniz FF, Greenbaum U, Pasvolsky O, Milton DR, Kanagal-Shamanna R, Ramdial J, et al. Characteristics and outcomes of patients with multiple myeloma who developed therapy-related acute myeloid leukemia and myelodysplastic syndrome after autologous cell transplantation. Transpl Cell Ther. 2024;30:205.e1–205.e12. 10.1016/j.jtct.2023.06.015 37437764

[16] Nadiminti K, Sidiqi MH, Meleveedu K, Alkhateeb HB, Hogan WJ, Litzow M, et al. Characteristics and outcomes of therapy-related myeloid neoplasms following autologous stem cell transplantation for multiple myeloma. Blood Cancer J. 2021;11:63. 33741897 10.1038/s41408-021-00454-y PMC7979889

[17] Arber DA, Orazi A, Hasserjian R, Thiele J, Borowitz MJ, Le Beau MM, et al. The 2016 revision to the World Health Organization classification of myeloid neoplasms and acute leukemia. Blood. 2016;127:2391–405. 27069254 10.1182/blood-2016-03-643544

[18] Mahindra A, Raval G, Mehta P, Brazauskas R, Zhang MJ, Zhong X, et al. New cancers after autotransplantations for multiple myeloma. Biol Blood Marrow Transpl. 2015;21:738–45. 10.1016/j.bbmt.2014.12.028 PMC4359647 25555448

[19] Richardson PG, Jacobus SJ, Weller EA, Hassoun H, Lonial S, Raje NS, et al. Triplet therapy, transplantation, and maintenance until progression in myeloma. N. Engl J Med. 2022;387:132–47. 35660812 10.1056/NEJMoa2204925 PMC10040899

[20] Govindarajan R, Jagannath S, Flick JT, Vesole DH, Sawyer J, Barlogie B, et al. Preceding standard therapy is the likely cause of MDS after autotransplants for multiple myeloma. Br J Haematol. 1996;95:349–53. 8904891 10.1046/j.1365-2141.1996.d01-1891.x

[21] Sperling AS, Guerra VA, Kennedy JA, Yan Y, Hsu JI, Wang F, et al. Lenalidomide promotes the development of TP53-mutated therapy-related myeloid neoplasms. Blood. 2022;140:1753–63. 35512188 10.1182/blood.2021014956 PMC9837415

[22] McCarthy PL, Owzar K, Hofmeister CC, Hurd DD, Hassoun H, Richardson PG, et al. Lenalidomide after stem-cell transplantation for multiple myeloma. N. Engl J Med. 2012;366:1770–81. 22571201 10.1056/NEJMoa1114083 PMC3744390

[23] Attal M, Lauwers-Cances V, Marit G, Caillot D, Moreau P, Facon T, et al. Lenalidomide maintenance after stem-cell transplantation for multiple myeloma. N. Engl J Med. 2012;366:1782–91. 22571202 10.1056/NEJMoa1114138

[24] McCarthy PL, Holstein SA, Petrucci MT, Richardson PG, Hulin C, Tosi P, et al. Lenalidomide maintenance after autologous stem-cell transplantation in newly diagnosed multiple myeloma: a meta-analysis. J Clin Oncol. 2017;35:3279–89. 28742454 10.1200/JCO.2017.72.6679 PMC5652871

[25] Elsallab, Ellithi M, Lunning MA M, D’Angelo, Ma C, Perales MA J, et al. Second primary malignancies after commercial CAR T-cell therapy: analysis of the FDA Adverse Events Reporting System. Blood. 2024;143:2099–105. 38483155 10.1182/blood.2024024166

[26] Ghilardi G, Fraietta JA, Gerson JN, Van Deerlin VM, Morrissette JJD, Caponetti GC, et al. T cell lymphoma and secondary primary malignancy risk after commercial CAR T cell therapy. Nat Med. 2024;30:984–9. 38266761 10.1038/s41591-024-02826-w

